# DEVELOPMENT OF THE ITALIAN VERSION OF THE MOTRICITY INDEX AND EVALUATION OF ITS RELIABILITY IN ADULTS WITH STROKE

**DOI:** 10.2340/jrm.v57.40441

**Published:** 2025-01-03

**Authors:** Diego LONGO, Stefano DORONZIO, Michele PIAZZINI, Angela Maria POLITI, Tommaso CIAPETTI, Filippo GERLI, Monica BARNABÉ, Francesca CIULLINI, Chiara CASTAGNOLI, Ilaria PELLEGRINI, Marta CANNOBIO, Donata BARDI, Marco BACCINI, Francesca CECCHI

**Affiliations:** 1 University of Florence, Department of Experimental and Clinical Medicine, Firenze, Italy; 2 IRCCS Fondazione Don Carlo Gnocchi, Firenze, Italy

**Keywords:** stroke, neurological rehabilitation, translation, reliability, outcome assessment

## Abstract

**Background:**

The Motricity Index (MI) is a commonly used method of measuring muscle strength in post-stroke hemiparesis. This study aimed to produce the MI Italian version (MI-IT) and assess its reliability in subjects with stroke.

**Methods:**

Phase-1: stepwise approach to MI-IT production and pilot-testing with 10 health professionals to ensure clarity of each item and instructions for administration and scoring. Phase-2: evaluation of MI-IT reliability on stroke subjects, each independently assessed by 2 raters randomly selected from a group of 10 physiotherapists; the first rater re-administered the MI-IT 1–3 days later. Intraclass correlation coefficients, Spearman’s rho and, limited to the more affected side, non-parametric limits of agreement (LOA) were computed for total MI-IT scores, squared weighted kappa and percentage of observed agreement for individual item scores.

**Results:**

The back-translated versions showed no discrepancies with original MI, but 3 items were revised after pilot-testing. Complete data on 50 (test–retest) and 51 (inter-rater) participants demonstrated excellent reliability of all MI-IT total scores on the more affected side (Spearman’s rho range: test–retest 0.953–0.975; inter-rater: 0.965–0.970), with LOA ranging from 9–25%), but poor inter-rater reliability for some scores on the less affected side (Spearman’s rho range: test–retest, 0.816–0.976; inter-rater: 0.508–0.721). Moderate to almost perfect agreement was found for all individual item scores, except for 2 items on the less affected side.

**Conclusions:**

The MI-IT is sufficiently reliable to evaluate motor impairment of the more affected side after stroke, with acceptable measurement error for all scores.

## LAY ABSTRACT

The Motricity Index (MI) is an evaluation tool widely used by physicians and physiotherapists to measure the severity of paralysis after brain injury. In this study, we translated the MI into Italian (MI-IT) using a rigorous procedure to ensure full correspondence to the original version, and the clarity of the translation was verified by ten Italian healthcare professionals. We then assessed the reliability of the MI-IT, i.e. the agreement between different examiners that use the MI-IT to independently assess the same person, or the variability of the measurement when the same examiner assesses an unchanged person twice. We enrolled a sample of 51 persons who had had a recent stroke and ten experienced physiotherapists as examiners, and found the MI-IT to have good overall reliability, with acceptable measurement error.

The Motricity Index (MI) is an ordinal method of measuring muscle strength in post-stroke hemiparesis developed by Demeurisse et al. (1) in 1980. Three movements for upper (pinch grip, PG; elbow flexion, EF; shoulder abduction, SA) and lower (ankle dorsiflexion, AD; knee extension, KE; hip flexion, HF) limb are assessed (1), using the Medical Research Council (MRC) 6-point scale (2), converted into modified weighted scores. A total score ranging from 0 (complete paresis) to 100 (normal strength) (3) is computed for each limb on both sides, and a side score can be computed averaging upper and lower limb scores. Administration time is 5–20 min, depending on the examiner’s experience and the severity of impairment (4).

MI is widely used and is recommended for clinical and research purposes for assessment of post-stroke patients at any stage and in any rehabilitation settings (5). The Italian Society of Physical and Rehabilitation Medicine (SIMFER) has included the MI in the Minimal Assessment Protocol for stroke survivors, termed PMIC2020 (6). The PMIC2020 has been developed to provide a minimal but comprehensive assessment to define needs and outcomes of the person with stroke throughout the rehabilitation process, from the acute and subacute phases to outpatient or home rehabilitation. However, only unofficial, non-validated Italian versions of MI are currently available. Even if the MI measures the strength of target muscles, and this parameter is presumably poorly related to the social-cultural context, a stringent translation procedure should still be carried out to ensure that the new language version is fully compliant with the original.

To ensure that the translated version is reliable it is important to evaluate whether the results are consistent among raters (inter-rater reliability) and can be reproduced for repeated measurements (test–retest reliability). Previous studies have evaluated the reliability only in small samples of persons with chronic (7) or subacute/chronic (3) stroke. Good to excellent test–retest and inter-rater reliability has been found (*r* ranging from 0.88–0.93), almost exclusively for the more affected side scores, and the measurement error estimated only for the more affected lower limb score (7). Thus, an in-depth and comprehensive assessment of the reliability of the MI is currently lacking, whichever language version is considered.

The aim of this study was therefore to develop an official Italian version of the MI (MI-IT) through a rigorous backward/forward translation process, to pilot-test the translated version in a sample of users to evaluate its clarity, and to assess its test–retest and inter-rater reliability in a sample of participants with stroke.

## METHODS

### Study design, setting and registration

This was a single-centre observational study conducted in accordance with COSMIN (Consensus-Based Standards for the Selection of Health Measurement Instruments) guidelines (8) at the scientific Institute Don Carlo Gnocchi Foundation, Florence, Italy (FDG). The study comprised 2 phases, i.e., translation of the MI into Italian, including pilot-testing to evaluate its clarity (phase_1), and initial evaluation of metric properties (test–retest and inter-rater reliability) (phase_2). The study was registered on ClinicalTrials.gov (registration number NCT05828160). On May 31, 2023, it was approved by the local Ethics Committee (registry number 22257_oss). Written informed consent was obtained for each subject enrolled.

### Procedures

**Fig. 1:**
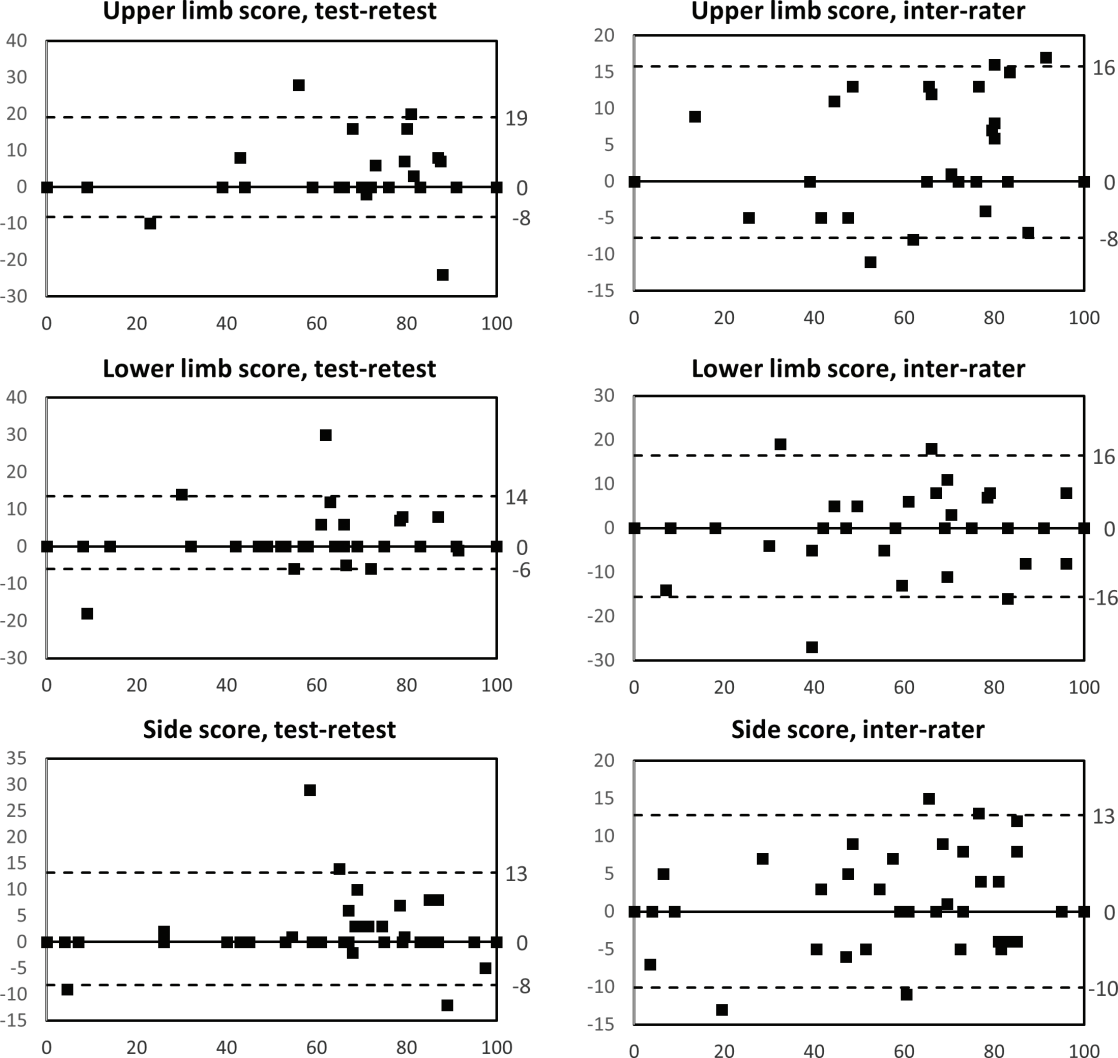
Bland–Altman plots showing the median of the differences and non-parametric limits of agreement for the more affected side MI-IT total scores.

*Phase 1: Development of MI-IT*. A comprehensive process involving forward/backward translations, multi-step revisions by different rehabilitation professionals and creation of preliminary and ultimate versions was conducted following international guidelines (Fig. 1) (9–11). Both the instrument and the instructions for administering and scoring each item and for total scoring, as reported by Collin and Wade (3), were included in the translation process.

The translation process was led by an interdisciplinary team assembled from the PROMISE@LAB at the FDG, including a physiatrist, 3 physiotherapists, a speech therapist, a psychologist, and an occupational therapist. All members possessed substantial expertise in stroke rehabilitation and research and were proficient in both Italian and English. Two qualified translators, neither of whom were familiar with the original MI or with Italian unofficial MI versions, independently translated the MI into Italian. The interdisciplinary team reviewed the 2 Italian versions and produced a single preliminary MI-IT, which was back-translated by 2 other qualified, native-English speakers translators blind to the original MI. The 2 back-translated versions were reviewed and compared with the original scale by the interdisciplinary team, joined at this stage by all the translators involved, who developed the pre-final MI-IT

The pre-final MI-IT was evaluated for clarity in a pilot study that involved 10 healthcare professionals (clinicians or physiotherapists) with at least 3 years’ experience in stroke rehabilitation, selected among employees at the FDG, none of whom had been part of the interdisciplinary team already described. They were asked to judge each MI-IT item (including scoring instructions) as “clear” or “unclear” and the percentage of “unclear” ratings was calculated. If it exceeded 20%, the item was revised in the final MI-IT (10).

*Phase 2: Reliability study. Participants*. Individuals with post-stroke hemiparesis were selected among patients referred from acute-care hospitals to the N2euromotor Rehabilitation Unit of FDG for intensive inpatient rehabilitation after stroke, where they remain on average 1 month (12). All potentially eligible patients were proposed to participate in the study by a member of the research team. Those who fulfilled the inclusion and exclusion criteria and signed an informed consent form were consecutively enrolled until the expected sample size was reached (*n* = 50, i.e., the minimum number for an “adequate” sample size according to COSMIN criteria (13). Inclusion criteria were: age > 18 years; presence of stroke outcomes impacting the person’s independence in activities of daily living, as measured by a modified Barthel Index score < 100; willingness to participate in the study. We did not include the level of motor impairment among eligibility criteria, because we aimed to enrol a sample in which all possible MI scores were represented. We also did not consider the time since stroke, although it was expected, given the setting, that participants were mostly in the subacute phase after stroke. The exclusion criteria were: severe and uncorrected visual/hearing impairment; cognitive/speech impairment hindering the comprehension and execution of the procedures included in the protocol; Clinical Instability Scale (CIS) score > 0 (14). The CIS assesses the presence of any changes in vital parameters and a CIS score = 0 indicates the reasonable medical probability, in accordance with recognized medical standards, that the patient’s condition will not materially deteriorate in the short term.

*Assessors*. Seventy pairs of raters (Rater1 and Rater2) were randomly drawn from a group of 10 physiotherapists with at least 3 years’ experience in post-stroke rehabilitation. Pairs were consecutively assigned to patients as they were enrolled. If a pair was unavailable at the time of assessment, the patient was assigned the next available pair. All evaluators had experience in administering MI-IT, but still participated in 2 x 1-hour training sessions to align in test administration and scoring. In particular, 2 points overlooked in the MI administration and scoring procedure described by Collin and Wade (3) were discussed and agreed upon by raters, i.e., the scoring when the joint passive range of motion was limited, and the possibility of verbally encouraging the participant during his/her attempt to perform the required movement. For both points, it was decided to use the criteria commonly adopted during manual muscle testing, so when the passive range of motion was limited, the active movement through the entire allowed range was considered as a complete movement; verbal encouragement was allowed if necessary.

*Procedure*. The 2 raters independently administered the MI-IT to the assigned subject, a short distance (30–60 min) apart, within 7 days from admission (TEST session). Rater1 (the first drawn when the pairs were formed) repeated the administration after 1 day (RETEST session), when possible, and in any case no later than 3 days after the test. Time for retest was chosen in order to minimize differences between the 2 moments of observation due to possible changes of functions in the subacute phase. All assessments with the MI-IT were performed directly in the participant’s hospital room, so that the setting was always the same. Raters were always blind to the other rater’s assessment and could not check their own previous findings.

The following data were collected: participant ID (consecutive recruitment number); stroke diagnosis and aetiology; age; schooling; gender; Mini Mental State Examination (MMSE) (15); CIS at recruitment (14); MI-IT at TEST (2 assessments by different raters) and RETEST (1 assessment by Rater1); National Institute Health Stroke Scale (NIHHS) (16); modified Rankin Scale (17); modified Barthel Index (18); and Fugl-Meyer Assessment Scale (19) at admission. Raters who administered the MI were blind to any clinical information concerning the evaluated subject, including all other clinical scales, which were administered by different examiners. The principal investigator was responsible for anonymous storage of all data in protected environments with limited access by the researchers.

*Data analysis*. For the total MI-IT scores (upper limb, lower limb, and side scores), the distribution of the data was preliminarily evaluated by means of the Shapiro–Wilk test, which showed that not all scores fitted into a normal distribution (always *p* < 0.001). Therefore, for these scores the Wilcoxon test was used to check for any significant differences between the 2 compared assessment sessions, which might have occurred as a learning/fatigue effect in assessments conducted a short time apart on the same day (Rater1–Rater2) or as the effect of an actual change in assessments conducted on different days (TEST–RETEST).

Reliability analysis of the total scores was estimated by the intraclass correlation coefficient (ICC1,1) and Spearman’s correlation coefficients. For scores of each MI-IT item, squared weighted kappa (wK) and percentages of absolute agreement were computed. For the interpretation of Spearman’s rho and ICC, we adopted the criteria proposed by Fitzpatrick et al. (20), who differentiated the requirements for using an instrument to measure groups or individual performance, and suggested reliability coefficients > 0.70 or > 0.90, respectively. We rated reliability as good if the first criterion was met, as excellent if the second was met, and as insufficient if neither was met. Agreement according to kappa values was interpreted as suggested by McHugh (21): 0–0.20 = none; 0.21–0.39 = minimal; 0.40–0.59 = weak; 0.60–0.79 = moderate; 0.80–0.90 = strong; > 0.90 = almost perfect.

Limited to the more affected side scores, we also estimated the measurement error using Bland Altman limits of agreement (LOA). Non-parametric limits (97.5^th^ and 2.5^th^ percentile) were used and the average bias was estimated as the median of the differences (computed as RETEST score – TEST score, or score of Rater 2 – score of Rater 1), as even paired differences were not normally distributed (22). We considered LOA to be excellent or acceptable when their percentage value – computed as (LOA/grand median) x 100 – was < 10° or between 10% and 30%, respectively, in line with what is commonly suggested for the minimal detectable change in rehabilitation studies (23, 24).

The statistical analysis was performed with jamovi (version 2.5) software (The jamovi project, 2024. Retrieved from https://www.jamovi.org).

## RESULTS

### Translation

When producing the preliminary MI-IT by comparing and reviewing the 2 independent Italian translations, the interdisciplinary team agreed on the following minor changes without altering the meaning of the text:

1. To have a person-centred focus, the word “patient”, which carries with it implications of passivity and is etymologically associated with patience and pain (25), was always replaced with “person”.
2. The wording “other side” was replaced with “contralateral” to use more technical language.
3. The different phrases used in the original MI to indicate full range of motion were always translated as “full range of movement”.
4. The verb “to place” has been substituted for “to be” when indicating the positioning of the small cube used for pinch assessment.
5. The wording “small hand muscles” was replaced with “intrinsic hand muscles” to use more technical language.
6. Similarly to other items, the movement instead of the joint was indicated in the item 2 heading: “Elbow flexion” instead of “Elbow”.
7. “Monitor biceps” and “monitor tibialis anterior” were changed to “monitor contraction of biceps/tibialis anterior” for consistency with the other items.
8. “Associated (trick) movement” was replaced with “associated compensatory movement”.
9. “Anterior thigh” was replaced with “anterior thigh surface”.
10. The sentence “when the hip is fully flexed, but easily pushed down” was converted to “when the hip is fully flexed, but the thigh can be easily pushed down”, because the segment, not the joint, is pushed down.

The comparison between the 2 back-translated versions and the original MI showed no semantic or conceptual discrepancy. Thus, the preliminary MI_IT was considered as the pre-final MI_IT, i.e., the version to be tested for comprehensibility in the pilot study.

### Pilot study on pre-final MI-IT

Seven physiotherapists and 3 medical doctors (3 men, 7 women; mean age 42.3 ± 11.6) were asked to judge the clarity of the pre-final MI-IT. Three points were unclear to more than 20% of the participants, so they were reviewed by the same interdisciplinary team involved in all the translation steps, to achieve the final MI-IT.

*1. Item 2 (EF)*. Some of participants argued that the translation into Italian of the sentence “The examiner may hold the elbow out so that the arm is horizontal” was somewhat ambiguous and did not indicate the exact positioning of the upper limb for this test. Therefore, the team decided to specify that the shoulder is positioned in abduction.

*2. Item 5 (KE), scoring*. The instructions for this item were judged unclear by most participants because they state to assign 14 points when less than half of full range is completed and 19 points when the movement is complete but cannot be performed against resistance. “What score is then given when the movement is more than half of full range but not complete?” The team considered the question well founded and decided that 14 points are assigned when knee extension is less than full range.

*3. Scoring*. The sentence “One point may be added to each limb score so that the top score is 100” was considered ambiguous because it seems to leave it up to the examiner to decide whether to add 1 point. The team decided to modify the instructions in this point stating that 1 point is added to each limb score, when score = 99.

The final MI-IT can be found in Supplementary material.

### Reliability of MI-IT

During the study period, lasting 18 months, we screened for eligibility 100 consecutive subjects with stroke admitted to the FDG. Twenty-seven patients did not meet the exclusion criteria because of clinical instability (15), cognitive deficits (9), or language barrier (3), whereas 18 refused to participate, leading to 55 patients enrolled (Table I). The RETEST was conducted on average 1.28 (0.66) days (range 1–3) after the TEST. Five subjects were not reassessed at RETEST by Rater1 within the time limit (3 days) and 4 subjects were not assessed by Rater2, due to personal circumstances or organizational problems. This resulted in a sample of 50 subjects for test–retest reliability and 51 subjects for inter-rater reliability.

**Table I:** Characteristics of participants

Variable	Values
Sex, *n* (%)	
Men	35 (63.6)
Women	20 (36.4)
Age (years), mean (SD), range	74.72 (12.43), 33–97
More affected side, *n* (%)	
Left	28 (50.9)
Right	26 (47.3)
Both	1 (1.8)
Days since stroke, mean (SD), range (SD), range	24.45 (20.89), 6–105
MMSE, mean (SD), range	23.77 (4.34), 14–30
mRS, mean (SD), range	3.77 (0.63), 2–5
mBI, mean (SD), range	51 (26.53), 6–94
NIHSS, mean (SD), range	6.32 (4.55), 0–17
FMA motor function, mean (SD), range	65.02 (32), 0–100
FMA sensory function, mean (SD), range	19.79 (6.23), 0–24

MMSE: Mini Mental State Examination; mRS: Modified Rankin Scale; mBI: modified Barthel Index; NIHSS: National Institute Health Stroke Scale; FMA: Fugl-Meyer Assessment Scale.

The 10 raters performed on average 10.7 ± 3.9 (range: 5–18) assessments in total, of which 5.4 ± 2.4 (range: 2–10) as Rater1 and 5.3 ± 2.9 (range: 2–11) as Rater2. Thus, all 10 raters participated in both the test–retest and inter-rater assessments for some participants.

As for the reliability of MI-IT total scores, no significant differences between assessments were found, except for the more affected upper limb score by different raters (Table II). On the more affected side, Spearman’s rho and ICCs ranged from 0.953–0.975 and 0.975–0.982, respectively (test–retest, Table II), and 0.965–0.970 and 0.947–0.980, respectively (inter-rater, Table III). On the less affected side, results were generally worse, particularly with regard to inter-rater reliability, where all Spearman’s coefficients were well below 0.90 and exceeded 0.70 only for the lower limb score. On the more affected side, the measurement error was always smaller for test–retest comparison than for inter-rater comparison, except for the upper limb score, with upper and lower LOA ranging from 13 to 19 and –6 to –16, respectively (Fig. 1).

**Table II:** Test–retest reliability of MI-IT total upper limb, lower limb and side scores

Factor	Test, median (IQR), min–max	Retest, median (IQR), min–max	*p*-value*	ICC (95%CI)	rho	Upper LOA (%)	Lower LOA (%)	Median of differences
MA_UL	74 (40.8), 0–100	76.0 (32.8), 0–100	0.068	0.975 (0.960–0.984)	0.953	19 (25)	–8 (11)	0
MA_LL	67.5 (43.5), 0–100	68.9 (41.3), 0–100	0.101	0.979 (0.967–0.987)	0.974	14 (20)	–6 (9)	0
MA_Side	69.5 (39.8), 0–100	73.5 (34.5), 0–100	0.064	0.982 (0.972–0.989)	0.975	13 (18)	–8 (11)	0
LA_UL	100 (0), 34–100	100 (0), 34–100	0.129	0.963 (0.942–0.977)	0.816	-	-	-
LA_LL	100 (9), 34–100	100 (9), 34–100	0.289	0.977 (0.963–0.985)	0.964	-	-	-
LA_Side	100 (7.2), 34–100	100 (5), 34–100	0.107	0.982 (0.972–0.989)	0.966	-	-	-

MA: more affected; LA: less affected; UL: upper limb; LL: lower limb; IQR: interquartile range; Min: minimum; Max: maximum; ICC: intraclass correlation coefficient; rho: Spearman’s correlation coefficient; LOA: limit of agreement.

* Wilcoxon test.

**Table III:** Inter-rater reliability of MI-IT total upper limb, lower limb, and side scores

Factor	Test, median (IQR), min–max	Retest, median (IQR), min–max	*p*-value*	ICC (95%CI)	rho	Upper LOA (%)	Lower LOA (%)	Median of differences
MA_UL	76 (46), 0–100	76.0 (47.5), 0–100	0.016	0.980 (0.969–0.988)	0.970	16 (21)	–8 (10)	0
MA_LL	68.9 (44), 0–100	71.9 (44), 0–100	0.819	0.947 (0.916–0.966)	0.965	16 (23)	–16 (22)	0
MA_Side	70 (39.5), 0–100	73 (34), 0–100	0.461	0.978 (0.965–0.986)	0.967	13 (17)	–13 (17)	0
LA_UL	100 (0), 37–100	100 (0), 37–100	1.000	0.886 (0.824–0.927)	0.508	-	-	-
LA_LL	100 (9), 37–100	100 (9), 37–100	0.532	0.908 (0.858–0.942)	0.721	-	-	-
LA_Side	100 (6.5), 37–100	100 (6.5), 37–100	0.752	0.913 (0.865–0.945)	0.699	-	-	-

MA: more affected; LA: less affected; UL: upper limb; LL: lower limb; IQR: interquartile range; Min: minimum; Max: maximum; ICC: intraclass correlation coefficient; rho: Spearman’s correlation coefficient; LOA: limit of agreement.

* Wilcoxon test.

The reliability of individual MI-IT item scores was variable, but generally better for the more affected side. On this side (Table IV), the test–retest k coefficients always exceeded 0.80, and were above 0.90 for 3 items (PG, AD, KE), whereas the inter-rater coefficients exceeded 0.90 only for PG and were between 0.60 and 0.80 for EF, SA KE, and HF. On the less affected side (Table V), the results were similar for test–retest reliability, but worse for inter-rater reliability, with k coefficients > 0.80 for only 2 items (AD, HF), and > 0.70 for only 2 other items (PG, SA). Despite the lower k coefficients, the observed agreement was always high on the less affected side, where it always exceeded 0.90, except on 2 items.

**Table IV:** Reliability of individual MI-IT item scores on the more affected limb

Factor	Assessment 1, median (IQR), min–max	Assessment 2, median (IQR), min–max	OA	wK (95% CI)
Inter-rater				
Pinch grip	26 (11), 0–33	26 (13.25), 0–33	0.78	0.979 (0.965–0.993)
Elbow flexion	25 (14), 0–33	25 (12.5), 0–33	0.72	0.773 (0.529–1.000)
Shoulder abduction	25 (15), 0–33	25 (19), 0–33	0.78	0.770 (0.571–0.969)
Ankle dorsiflexion	25 (19), 0–33	25 (19), 0–33	0.76	0.821 (0.615–1.000)
Knee extension	25 (19), 0–33	25 (19), 0–33	0.72	0.778 (0.594–0.962)
Hip flexion	25 (11), 0–33	22 (11), 0–33	0.68	0.638 (0.400–0.876)
Test–retest				
Pinch grip	26 (11), 0–33	26 (11), 0–33	0.84	0.956 (0.911–1.000)
Elbow flexion	25 (14), 0–33	25 (8), 0–33	0.84	0.850 (0.678–1.000)
Shoulder abduction	25 (15), 0–33	25 (19), 0–33	0.77	0.864 (0.715–1.000)
Ankle dorsiflexion	25 (19), 0–33	25 (19), 0–33	0.84	0.918 (0.831–1.000)
Knee extension	25 (19), 0–33	25 (19), 0–33	0.88	0.956 (0.909–1.000)
Hip flexion	25 (11), 0–33	25 (11), 0–33	0.80	0.833 (0.643–1.000)

wK: weighted kappa (squared); OA: percentage of observed agreement.

**Table V:** Reliability of individual MI-IT item scores on the less affected limb

Factor	Assessment 1, median (IQR), min–max	Assessment 2, median (IQR), min–max	OA	wK (95% CI)
Inter-rater				
Pinch grip	33 (0), 11–33	33 (0), 11–33	0.96	0.793 (0.398–1.000)
Elbow flexion	33 (0), 9–33	33 (0), 9–33	0.92	0.500 (0.075–0.925)
Shoulder abduction	33 (0), 14–33	33 (0), 14–33	0.90	0.773 (0.477–1.000)
Ankle dorsiflexion	33 (0), 9–33	33 (0), 0–33	0.90	0.817 (0.639–0.995)
Knee extension	33 (0), 0–33	33 (0), 9–33	0.88	0.381 (0.005–0.758)
Hip flexion	33 (8), 0–33	33 (0), 0–33	0.80	0.873 (0.748–0.998)
Test–retest				
Pinch grip	33 (0), 11–33	33 (0), 11–33	0.98	0.886 (0.607–1.000)
Elbow flexion	33 (0), 9–33	33 (0), 9–33	1.00	1.000 (1.000–1.000)
Shoulder abduction	33 (0), 14–33	33 (0), 14–33	0.90	0.871 (0.715–1.000)
Ankle dorsiflexion	33 (0), 9–33	33 (0), 0–33	0.92	0.850 (–0.400–1.000)
Knee extension	33 (0), 0–33	33 (0), 0–33	0.96	0.911 (–0.465–1.000)
Hip flexion	33 (8), 0–33	33 (8), 0–33	0.96	0.973 (0.931–1.000)

wK: weighted kappa (squared); OA: percentage of observed agreement.

## DISCUSSION

In the present study, we developed an official version of the MI and preliminarily tested its reliability in a sample of participants with subacute stroke. We found good to excellent test–retest reliability for all total scores and excellent inter-rater reliability for more affected side scores, but good or insufficient inter-rater reliability for less affected side scores. On the more affected side, however, despite the high correlation coefficients, the measurement error was far from negligible for most scores, as the LOA always exceeded 10%, with the exception of the intra-rater lower LOA of the lower limb score.

The absence of an official Italian version of MI so far is likely be due to the function assessed by the scale, which largely corresponds to manual muscle testing procedures, and as such does not require cross-cultural adaptation. Nevertheless, the importance of a rigorous approach to translation is highlighted by the results of the pilot study on the clarity of the pre-final MI-IT. In fact, criticism on some items from professionals was instrumental to produce the final MI-IT, in which some minor inaccuracies were corrected.

This is the first study that has comprehensively addressed the reliability of MI. With regard to total scores, test–retest and inter-rater reliability of the more affected side scores may be rated as excellent, because it is high enough to use the scale for making decisions on individuals. The difference between the 2 raters for the upper limb score, although significant, was still very small (on average 2.2 points), so we do not believe it had a major impact on reliability. However, analysis of the measurement error induces some caution, as a variability between 17% to 25% more and 9% to 22% less is expected, depending on the score.

Reliability of the less affected side scores was generally worse. With regard to inter-rater reliability in particular, the coefficient does not even reach the minimum acceptable value for evaluations at group level for the upper limb score, and barely reaches or exceeds it for lower limb and side scores. The reduced inter-rater reliability is likely due to the fact that, on this side, the muscle strength was most often normal or near normal. In these cases, it is not possible to use an objective criterion for grading the strength (e.g., the range of active movement), which is awarded on the basis of the resistance that the subject can overcome; but when assessing the less affected side, the examiner cannot use a “healthy” contralateral limb for comparison. Indeed, current evidence indicates that manual muscle testing scores, based on the 0–5 point MRC scale (the base for MI scoring), must change more than 1 full grade to be confident that a true difference exists (26). We decided not to compute LOA for the less affected side scores, because the vast majority of subjects had no apparent weakness and scored highest on this side.

Results are in line or even better than previous studies that explored MI reliability. Collin and Wade (3) reported, in 20 stroke patients, Spearman’s coefficients of 0.88, 0.87, and 0.88 for upper limb, lower limb, and side score, respectively (likely of the more affected side, although not explicitly declared). In a separate publication by the same group, test–retest reliability of lower limb scores over a span of 2–3 weeks was assessed in 20 chronic stroke survivors (4), but only the differences between the first and second assessment, cumulatively for less affected and more affected side, are reported. From the data presented by the authors, we computed non-parametric upper and lower LOA of 18 and –23, respectively, which are higher than what was found in the present study. A third study (7) assessed only test–retest reliability of the more affected lower limb scores in 20 stroke survivors, finding an ICC of 0.93, slightly worse than ours, and a standard error of measurement of 4.66. The latter value results in a minimal detectable change of about 13 points, which is higher than the range of error we estimated using the LOA (14; –6). It is noteworthy that Fayazi et al. (7) enrolled mostly participants with chronic stroke, whereas all our participants were in the subacute phase. As results are consistent, we may conclude that MI-IT appears to be just as reliable when administered shortly after the acute event as in more chronic phases.

As for individual MI item scores, no studies have investigated the reliability of each MI item so far. On the more affected side, we found test–retest kappa coefficients interpretable as having almost perfect (PG, AD, KE) or strong (EF, SA, HF) agreement; conversely, inter-rater agreement was almost perfect and strong in 1 case each (PG and AD, respectively), and moderate for the other items. HF showed the lowest agreement, both test–retest (wK = 0.833) and inter-rater (wK = 0.638), which may depend on several reasons: first, defining the full range of motion, which is greatly affected by pelvic rotation (27), may be difficult, as well detecting compensations (backward trunk movement), particularly in patients with recent stroke who may have problems with balance when sitting; the weakness of muscles that are activated as pelvic stabilizers for this movement also impact the ability to complete the movement; moreover, if the patient is unable to sit, the test is performed in the supine position, but the effect of gravity is quite different and examiners lose some objective criteria for grading. All these features also impact the other movements assessed, but to a much lesser extent.

On the less affected side, test–retest reliability was comparable to the more affected side (almost perfect agreement for EF, KE, HF; strong agreement for PG, SA, AD). In contrast, inter-rater kappa values indicated substantial agreement for AD and HF, moderate agreement for PG and SA, weak agreement for EF, and minimal agreement for KE. In both the last 2 cases, however, the observed agreement was near to or even above 90%, just the same or even higher than the observed agreement found for the other items. This conflict between observed agreement and kappa values is most likely due to the well-known phenomenon of kappa paradox (28), which also occurs when percentages of agreement/disagreement on different scores are highly unbalanced, as happens in the less affected limbs, where only a few participants exhibited some degrees of muscle weakness. In such cases, the agreement due to chance is very high, and even a few instances of disagreement can drastically reduce the kappa coefficient, which then fails to reflect true agreement.

This study overcomes some limitations of current literature, which consists in underpowered studies where a single evaluator (7) or the same 2 evaluators (3,4) assessed all study participants. In the present work, 10 raters were involved, with random pairs assigned to each participant, and all acted as first or second rater for some subjects; therefore, although each participant was evaluated by a single rater for test–retest reliability, and by only 2 raters for inter-rater reliability, our data came from 10 raters and not just 1. Moreover, MI-IT reliability was tested in a sample size considered adequate according to COSMIN criteria. For these reasons, we believe that our results can be generalized to this population – adults with recent stroke – and to these evaluators – physiotherapists experienced in stroke rehabilitation who participated in 2 x 1-hour training sessions on MI administration.

Limitations of this study may lie in the selection process. Despite no limits being set as regards the time distance from stroke, the actual sample was composed only of subjects in the subacute phase. However, this might conceivably have led to underestimation, rather than overestimation, of MI reliability, as discussed earlier. Moreover, the MI is mainly used in this phase, so it was important to verify whether it is reliable in this population. On the other hand, enrolling participants in the subacute phase after stroke forced us to perform the retest evaluation with a very short time interval after the test in order to limit the occurrence of actual changes. This choice increases the possibility of overestimating test–retest reliability due to recall bias. However, it is conceivable that this bias had a minor effect, because the assessors usually evaluated more than 1 patient in a day with different tools and remembering all the MI scores assigned to patients (12 different scores, 3 for each limb) would have been difficult. Another limitation might be that about half of the eligible subjects (*n* = 45) were not included because they were unwilling to participate (*n* = 18) or were excluded due to clinical instability or cognitive/communication disorders (*n* = 27), but the latter is indeed an intrinsic limit of the scale.

The present study has some relevant clinical implications. In the MI-IT, a few minor inconsistencies and unclear points on the administration and scoring of some items have been corrected, so that rehabilitation professionals in Italy will now be able to use this instrument following a well-defined standardized procedure. Moreover, the data presented indicate that the MI-IT is sufficient reliable to evaluate the motor impairment on the more affected side in subjects who had a recent stroke. However, an error of 17% to 25% more, and 9% to 23% less can be expected, depending on the score considered, so rehabilitation professionals are now aware that only a variation above these values can be confidently considered indicative of a real change. Further research is needed to verify the results in larger samples also enrolling subjects with chronic stroke, and to investigate MI validity and responsiveness.
